# When Wernicke’s encephalopathy mimics stroke: A case report of atypical thalamic involvement

**DOI:** 10.1016/j.ibneur.2025.05.006

**Published:** 2025-05-25

**Authors:** Govind Singh Mann, Neeti Ajay Gupta, Nitin Jain

**Affiliations:** a Department of Neurology, Sant Parmanand Hospital, Delhi, India; b Department of Radiology, Sant Parmanand Hospital, Delhi, India

**Keywords:** Thiamine, Metabolic, Stroke, Thalamus, MRI, Ataxia, Oculomotor, Encephalopathy

## Abstract

Wernicke’s Encephalopathy (WE) is a metabolic disorder caused by thiamine deficiency, most commonly linked to chronic alcohol use. It typically presents with a triad of ataxia, mental confusion, and oculomotor abnormalities, with classic MRI findings showing symmetric hyperintensities in the mammillary bodies, thalamus, and periaqueductal region. The authors report a case of a 55-year-old male with WE presenting atypical unilateral thalamic hyperintensities on MRI, initially misdiagnosed as ischemic stroke. Delayed thiamine supplementation led to significant clinical improvement and resolution of atypical findings, with subsequent emergence of traditional bilateral WE changes on MRI. This case emphasizes the importance of early thiamine therapy and demonstrates that treatment can still be beneficial even when delayed.

## Highlights

• A rare case of WE with isolated thalamic hyperintensity on MRI, mimicking an ischemic stroke.
• Early thiamine led to significant clinical and radiological improvement in this patient.
• Imaging evolved from unilateral thalamic to classical bilateral WE pattern over time.
• The case stresses detailed alcohol history in atypical neuroimaging presentation of WE.

## Introduction

Wernicke’s Encephalopathy (WE) is a central nervous system (CNS) metabolic disorder caused by vitamin B1 deficiency. In the general population, the prevalence of WE has been estimated to be 0.4–2.8 %, with a female-to-male ratio of 1:1.7 (Chandrakumar and Bhardwaj, 2019). WE is mainly induced by malnutrition due to alcohol dependence (Freund, 1973). It is characterized by ataxia, mental confusion, and oculomotor disorders such as ophthalmoplegia and nystagmus (Kattah, 2017). Magnetic resonance imaging (MRI) of the brain shows symmetrical involvement in areas of rapid glucose metabolism, such as the mammillary bodies, thalamus, periaqueductal gray matter, and around the third ventricle (Ota et al., 2020).

The authors present a case involving a 55-year-old male who exhibited an acute onset of confusion, vertical gaze palsy, bilateral low-frequency tremors, right-sided cerebellar ataxia, and instability in gait characterized by a tendency to fall toward the right side. He was initially misdiagnosed as acute stroke, with MRI of the brain revealing unilateral involvement of the thalamus. The patient showed no clinical response to thrombolytics, and instead showed significant improvement following Thiamine supplementation. This case report represents the first documented instance of unilateral MRI findings during the acute phase of WE. Follow-up imaging performed after a few days revealed the traditional involvement of the mammillary bodies, thalamus, and periaqueductal white matter.

## Case presentation

A 55-year-old male patient presented to the Neurology Outpatient Department (OPD) with a history of acute onset of visual blurring, confusion, short-term memory impairment, difficulty in speech, and unsteadiness, manifesting approximately 20 days prior. The onset of symptoms occurred suddenly during dinner, prompting the patient to seek immediate medical attention at a tertiary care hospital where he was advised to undergo a non-contrast computed tomography (NCCT) of the head, a procedure he declined. Six days later, the patient developed gait imbalance and slurred speech, which prompted him to consult a physician at a local clinic. The clinician recommended an urgent magnetic resonance imaging (MRI) of the brain, which the patient subsequently underwent at a nearby diagnostic center. The imaging results indicated an initial diagnosis of ischemic stroke, and the patient was initiated on aspirin therapy. However, after two weeks, the patient experienced further deterioration of his symptoms, which led him to come to our Neurology OPD with above presenting complaints. During this visit, the patient disclosed a history of chronic alcohol consumption over the past 20 years; he reported that he had abstained from alcohol intake approximately one month prior to his presentation.

The general examination yielded unremarkable findings. The neurological assessment indicated disorientation to time, while the patient remained oriented to place and person with notable findings including vertical gaze restriction, bilateral low-frequency hand tremors with right side predominance, an ataxic gait with a tendency to fall towards the right side, and positive results on the finger-nose-finger test (right side) and heel-to-shin test (right side). Further evaluations, including cardiology, pulmonary, and abdominal examinations, were unremarkable. The patient was from a poor socio-economic background, therefore was managed on OPD-basis.

**Fig. 1:**
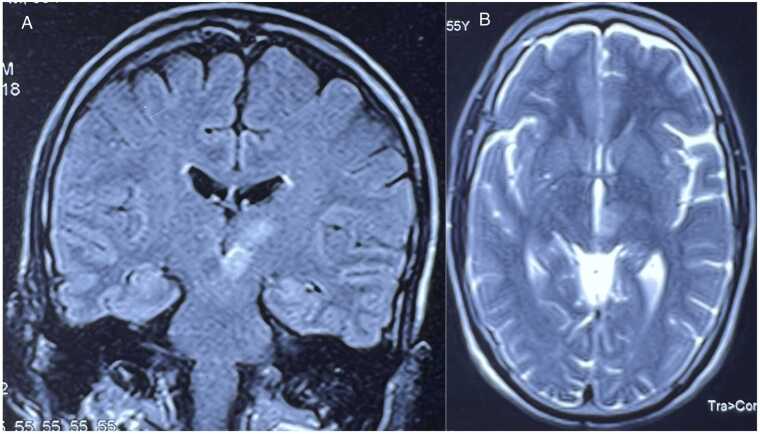
Coronal T2 FLAIR (A) and axial T2W (B) MR image showing hyperintensity involving the medial aspect of left thalamus and left cerebral peduncle.

**Fig. 2:**
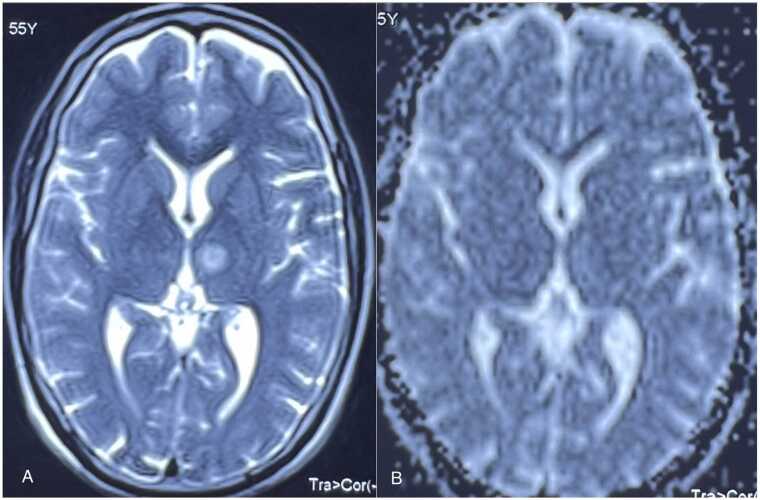
Axial T2-weighted (A) MR images showing a focal hyperintense area in medial aspect of left thalamus, which shows no signal drop on the corresponding ADC map (B).

Routine laboratory investigations indicated an elevated triglyceride levels of 152 mg/dL, an increased low-density lipoprotein level of 106 mg/dL, and elevated serum glutamic-oxaloacetic transaminase (SGOT) at 49 IU/L, along with serum glutamic-pyruvic transaminase (SGPT) at 71.7 IU/L. Serum thiamine levels could not be obtained due to limited laboratory availability and the high cost of testing, which were significant considerations given the patient’s socioeconomic constraints. Initial MRI brain obtained from the diagnostic center showed altered signal intensity areas appearing hyperintense on T2W/Fluid attenuated inversion recovery (FLAIR) images, showing no diffusion restriction on diffusion weighted imaging (DWI) or blooming on gradient sequence, as shown in Fig. 1 and Fig. 2 involving the medial aspect of left thalamus, extending inferiorly into the left crus cerebri. The mammillary bodies and right thalamus showed no significant signal abnormality. These MRI findings were notable for their unilateral distribution and interestingly, they corresponded well with the patient’s unilateral presentation. Clinical suspicion of Wernicke’s Encephalopathy prompted the initiation of conservative management accompanied by thiamine supplementation. Both Electroencephalogram (EEG) and 2-dimensional echocardiography were reported as normal.

**Fig. 3:**
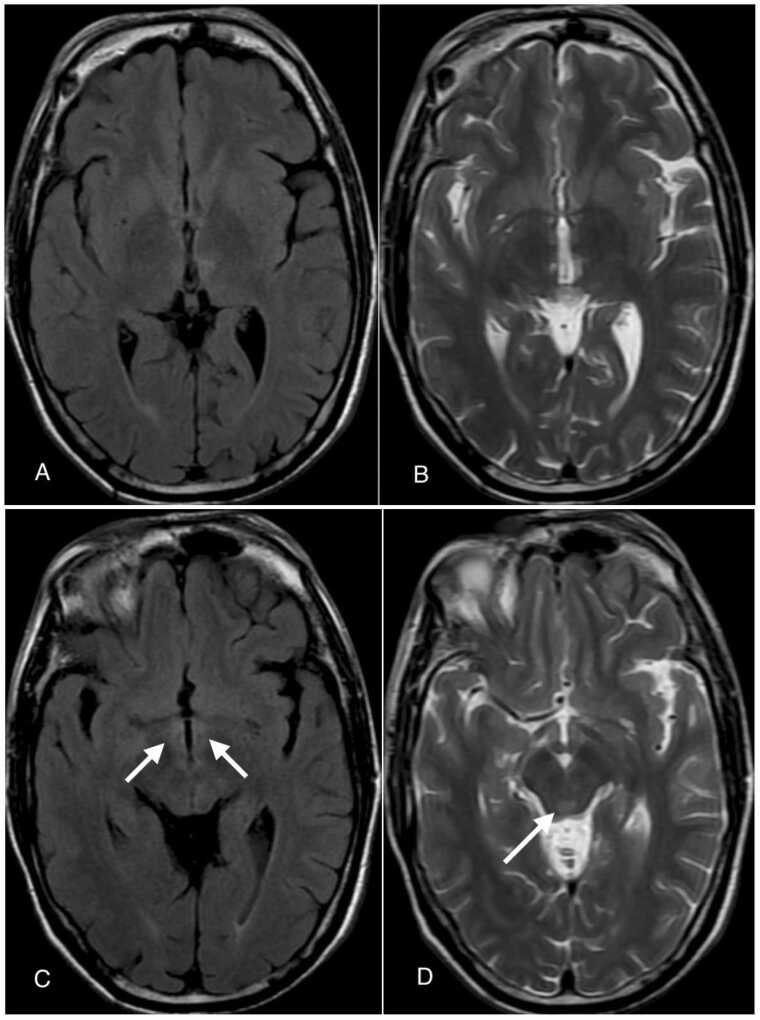
Axial T2-FLAIR (A and C) and T2-weighted (B and D) images showing decrease in the size of the previously seen altered signal intensity areas involving left thalamus and left cerebral peduncle with minimal T2 Flair hyperintensity seen in medial aspects of both thalami, mammillary bodies and in the periaqueductal region (white arrows in C and D).

In the next couple of days, the patient came back to the OPD and exhibited minor clinical improvement in both speech and gait, with the ability to ambulate independently. In the following days, further advancements were noted over the call in terms of his speech and cognitive functions. After 2 weeks of presentation to the OPD, the patient demonstrated significant clinical improvement with mild blurring of vision, a repeat MRI of the brain was performed which showed significant decrease in the size of altered signal intensity areas compared to previously seen altered signal intensity areas involving the left thalamus and left cerebral peduncle, with minimal T2 FLAIR hyperintensity seen in medial aspects of both thalami, mammillary bodies, and in the periaqueductal white matter as shown in Fig. 3. Notably, there were no signal changes observed around the fourth ventricle or cerebellum on the second MRI to account for the patient’s right-sided ataxia and gait disturbance. The patient is currently under follow-up, continuing to show clinical improvement alongside routine laboratory investigations, with notable resolution of the previously observed vertical gaze restriction and nystagmus, and no emergence of new ocular abnormalities following the subsidence of initial symptoms.

## Discussion

WE is a potentially life-threatening yet reversible neurological disorder that arises from thiamine deficiency. The classical triad of symptoms occurs in approximately one-third of affected individuals, but since many patients present with a wide range of non-specific clinical features, it often leads to misdiagnosis (Sinha et al., 2019). In this context, Caine’s operational criteria have been proposed to improve diagnostic sensitivity, requiring two of the following four features: dietary deficiency, oculomotor abnormalities, cerebellar dysfunction, and either altered mental status or mild memory impairment (Caine et al., 1997). WE can be broadly categorized into alcoholic and non-alcoholic forms, based on the underlying etiology. It is more frequently associated with severe alcohol use disorder, which results in diminished intestinal thiamine absorption. Conversely, non-alcoholic causes may include poor nutritional status, chemotherapy, severe vomiting, AIDS, gastrointestinal fistula, and bariatric surgery (Habas et al., 2023).

Thiamine is a crucial vitamin involved in mitochondrial energy metabolism and osmotic regulation within the CNS. Its deficiency may manifest as vestibular and oculomotor abnormalities, including upbeat nystagmus and gaze palsies, due to selective vulnerability of brainstem and cerebellar circuits (Kattah, 2017, Ota et al., 2020). MRI findings characteristic of WE include hyperintense signals on T2-weighted and FLAIR images in the mammillary bodies, thalamus, tectal plates, and periaqueductal regions (Ota et al., 2020).

The triad of WE encompasses cerebellar dysfunction, changes in mental status, and oculomotor disorders. Among the mental status changes, confusion, memory impairment, spatial disorientation, and cognitive deficits are the most prevalent symptoms (Ota et al., 2020). Ocular abnormalities, particularly horizontal nystagmus and gaze restrictions as a result of cranial nerve palsies, are commonly observed, while complete ophthalmoplegia remains rare. Cerebellar dysfunction frequently manifests as gait ataxia (Manzo et al., 2014).

In this case the patient is presented with acute cerebellar symptoms predominantly affecting the right side with decreased cognitive function and oculomotor symptoms. The patient's initial MRI was surprising as it showed only left thalamic altered signal intensity which favored acute ischemic event, but it was eventually ruled out with no supporting findings on DWI. Another noteworthy factor was the history of chronic alcohol consumption, due to which thiamine supplementation was implemented, which not only improved the patient, but also radiologically, thus proving that the previous MRI changes were in fact secondary to an atypical imaging presentation of WE. In addition to the imaging, the clinical findings were also notably unilateral with right-sided cerebellar ataxia and dysmetria aligning contralaterally to the left thalamic and peduncular lesions. This reinforces the atypical nature of the presentation and supports the diagnosis of unilateral WE both clinically and radiologically.

## Conclusion

This case highlights an atypical presentation of WE with unilateral thalamic involvement on MRI during the acute phase, which is a rare manifestation and previously undocumented occurrence in WE cases. The patient’s history of chronic alcohol use, combined with initial misdiagnosis as an ischemic event, underscores the critical importance of thorough alcohol consumption history and clinical suspicion for WE. Early thiamine supplementation not only resolved the symptoms but also normalized radiological abnormalities, thus documenting the diagnostic and therapeutic value of thiamine supplementation. The case illustrates the potential for unilateral involvement at the onset of WE and serves as an important reminder for healthcare providers regarding the timely identification and initiation of thiamine treatment, regardless of the duration of symptoms, to prevent life-threatening complications.

## Ethical Approval

Not applicable

## Funding Declaration

No funding was received for this report

## Informed Consent

The patient provided written informed consent for the publication of this case report. All identifying information was removed to maintain anonymity and confidentiality.

## CRediT authorship contribution statement

**Nitin Jain:** Writing – review & editing, Validation, Project administration, Formal analysis. **Neeti Ajay Gupta:** Writing – review & editing, Writing – original draft, Methodology, Formal analysis. **Govind Singh Mann:** Writing – review & editing, Writing – original draft, Visualization, Resources, Investigation, Data curation.

## Declaration of Competing Interest

The authors declare that they have no known competing financial interests or personal relationships that could have appeared to influence the work reported in this paper.
